# Evaluation of the efficacy of an interdialytic “ethanol 40% v/v - enoxaparin 1000 U/mL” lock solution to prevent tunnelled catheter infections in chronic hemodialysis patients: a multi-centre, randomized, single blind, parallel group study

**DOI:** 10.1186/s12882-019-1338-6

**Published:** 2019-04-30

**Authors:** Julien Aniort, Aurélien Piraud, Mireille Adda, Bruno Perreira, Marc Bouiller, Jacques Fourcade, Abdallah Guerraoui, Emilie Kalbacher, Thierry Krumel, Hélène Leray Moragues, Damien Thibaudin, Carlos Gustavo Vela, Guillaume Vernin, Hugo Weclawiak, Lise Bernard, Anne Elisabeth Heng, Bertrand Souweine

**Affiliations:** 1 Nephrology, Dialysis and Transplantation Department, Gabriel Montpied University Hospital, 54 rue Montalembert, BP69, 63003 Clermont-Ferrand, Cedex 1, France; 2 Medical Intensive Care Unit, Gabriel Montpied University Hospital, Clermont-Ferrand, France; 3 Biostatistics Unit (DRCI), University Hospital of Clermont-Ferrand, Clermont-Ferrand, France; 4 Nephrology and Dialysis Department, Emile Roux Hospital, Le Puy en Velay, France; 5 Nephrology and Dialysis Department, Metropole-Savoie Hospital, Chambery, France; 6 Calydial Viennes, Vienne, France; 7 Nephrology and Dialysis Department, Edouard Herriot University Hospital, Lyon, France; 8 Nephrology and Dialysis Department, University Hospital, Strasbourg, France; 9 AIDER, Montpellier, France; 10 Nephrology and Dialysis Department, University Hospital, Saint Etienne, France; 11 Nephrology and Dialysis Department, Hospital, Perpignan, France; 12 AGDUC, La Tronche, France; 13 Nephrology and Dialysis Department, Médipole Saint-Roch Clinic, Cabestany, France; 14 Pharmacy department, Gabriel Montpied University Hospital, Clermont-Ferrand, France; 15 CNRS UMR 6023, Laboratoire Microorganismes: Génome et Environnement, Université Clermont-Auvergne, Clermont-Ferrand, France

**Keywords:** Tunnelled, Hemodialysis, Catheter, Infection, Ethanol, Low molecular weight heparin, Lock

## Abstract

**Background:**

Tunnelled dialysis catheter (TC) infections are a major health complication and are associated with increased antibiotic consumption, hospital stays, health costs and mortality. Experimental data provide evidence that Ethenox, a mixture of enoxaparine 1000 U/mL in 40% *v*/v ethanol, could be a promising lock solution. The aim of the study is to compare an interdialytic lock solution of Ethenox with reference lock solutions, unfractionated heparin (UFH) or citrate 4% for the prevention of TCI in hemodialysis patients.

**Method:**

This study will monitor a multicentre, prospective, single blind, randomized, controlled, parallel group trial. The main inclusion criteria are patients > 18 years old with end-stage renal disease, treated with chronic hemodialysis/hemodiafiltration three times a week, with incident or prevalent non-impregnated internal jugular TCs inserted for at least 2 weeks and able to give informed consent. Exclusion criteria are TCI in the previous 4 weeks and anti-infective treatment for TCI in the previous 2 weeks. Patients will be randomized to receive either study treatment Ethenox in the intervention group or reference solutions in the control group, unfractionated heparin (UFH) or citrate 4% *w*/*v* according to usual practice. The primary outcome measure will be time to first TCIs assessed by an endpoint adjudication committee blinded to the study arm according to predefined criteria. Patients will receive the study treatment for up to 12 months. Intention-to-treat analysis of the primary endpoint will be performed with a marginal Cox proportional hazard model. Prospective power calculations indicate that the study will have 90% statistical power to detect a clinical significant two-fold increase in median infection-free survival if 200 patients are recruited into each arm over a period of 24 months.

**Discussion:**

Firm evidence of the efficacy of the Ethenox lock in preventing TCI could be of major clinical benefit for patients. The results of this study will allow the development of new guidelines based on a high level of evidence.

**Trial registration:**

ClinicalTrials.gov Identifier: NCT03083184, date of registration March 17 2017 and European Clinical Trials Database Identifier: EudraCT 2016-A00180-51), date of registration July 11 2016.

**Electronic supplementary material:**

The online version of this article (10.1186/s12882-019-1338-6) contains supplementary material, which is available to authorized users.

## Background

Infections are the second cause of death in end-stage renal disease patients treated by hemodialysis. The risk of infection of vascular access is ten times greater in chronic hemodialysis patients with a tunnelled catheter (TC) than in those with an arteriovenous fistula. In patients with TC infection (TCI), bacteremia and secondary septic foci occur in 3 to 44% of cases [1]. In chronic hemodialysis patients, TCI results in antibiotics prescription, frequent hospitalizations, and high health costs, and reduces survival [2, 3]. Thus, the prevention of TCI is a major means of improving the prognosis of hemodialysis patients.

Unfractionated heparin (UFH), is the standard interdialytic lock solution for the prevention of TC thrombosis. However, UFH has no antibiofilm properties and could even promote bacteria development [4]. Antibiotic locks decrease the rate of TCI including TC-related bloodstream infections (TCBSI) and exit-site infection (ESI). The widespread use of antibiotic lock solutions raises concerns about the development of adverse events and the emergence of antimicrobial resistance [5, 6]. Antiseptic locks could help to overcome the problem of acquired antibiotic resistance. Hypertonic citrate lock solutions were initially shown to reduce the risk of TCBSI [7, 8]. However, they lead to increased risk of severe pulmonary embolism and even cardiac arrhythmia due to hypocalcaemia, probably as the result of systemic diffusion of concentrated citrate [9]. Other studies found no significant difference in TCBSI incidence between citrate 4% *w*/*v* and UFH lock [10, 11]. A recent meta-analysis reported that antimicrobial-containing citrate locks but not citrate alone reduce the risk of TCI [12]. The use of taurolidine-citrate 4% w/v locks reduces the rate of TCI but increases that of TC dysfunction and TC removal for occlusion [13]. When taurolidine citrate is combined with UFH twice weekly and urokinase once weekly, TC dysfunction does not increase relative to UFH alone. However, the decrease in TCIs caused by Gram-positive cocci, which are the most prevalent cause of TCI in France, is not significant [14]. Hence, it is necessary to develop alternative lock solutions containing an antithrombotic and an additional non-antibiotic substance with antibiofilm properties.

Ethanol is an inexpensive antiseptic agent with activity against a broad range of bacteria and fungi commonly involved in TCI. It acts by non-specific protein denaturation and thus is less likely to promote antimicrobial resistance. Ethanol concentration ≥ 40% *v*/v is highly effective in eradicating biofilm of *Staphylococcus aureus*, *Staphylococcus epidermidis*, *Pseudomonas aeruginosa*, *Klebsiella pneumoniae* and *Candida albicans* [15–17] whereas 46.7% citrate has no antibiofilm properties [18]. Studies have been performed on the integrity of TCs exposed to ethanol: several showed that prolonged exposure of polyurethane and silicon TCs to ethanol at 60% v/v and 70% v/v does not cause ultra-structural damage (electron microscopy studies) or modify their mechanical properties [19, 20]. Likewise, the risk of releasing substances from silicon [21] and polyurethane [22] TCs, as assessed by mass spectrometry, is low at concentrations of ethanol below 60% *v*/v and not different from that observed with a sodium chloride solution at 0.9%.

The HEALTHY-CATH study [23], involving 49 hemodialysis patients fitted with a TC, is the only randomized open label controlled trial to have assessed the efficacy of an ethanol lock strategy for preventing TCBSIs. In the intervention group a-70% *v*/v ethanol interdialytic lock solution was instilled once a week. The reduction of TCBSI risk in this group was not significantly lower than in the heparin group, a result that could have been due to the insufficient power generated by the small patient sample. More recently, a meta-analysis showed that ethanol lock conferred significant benefit in preventing catheter-related bacteraemia in patients with a TC [24].These results suggest that ethanol lock is effective in reducing the rate of TCBSI.

However, ethanol has no specific anticoagulant effect, and our group has previously reported an increase in the rates of TC dysfunction when a 60% *v*/v ethanol interdialytic lock solution is used [25]. Experimental data provide evidence that a mixture of ethanol and injectable anticoagulant may be a promising lock solution for preventing both TCI and TC dysfunctions. UFH, whatever its concentration, cannot be mixed in 40% v/v ethanol because of precipitation. ERA-EDTA recommends using low molecular weight heparins (LMWHs) for blood circuit anticoagulation during dialysis sessions. Like UFH, LMWHs have no antibiofilm properties. In contrast, they can be mixed in 40% v/v ethanol and among LMWHs enoxaparin has the highest solubility (up to 1300 U/mL). Our group has shown that 40% v/v ethanol - enoxaparin 400 U/mL is stable, compatible with TC materials and possesses antibiofilm and anticoagulant properties in vitro (patent). No clinical studies have previously assessed the efficacy of a combined solution of ethanol and LMWH in preventing TCI in chronic hemodialysis patients.

We assume that an interdialytic lock solution of Ethenox, a mixture of enoxaparin 1000 U/mL in 40% v/v ethanol, can be effectively and safely used to prevent TCI in chronic hemodialysis patients. The reduced concentration of ethanol in Ethenox compared to that in previous published reports and the aspiration of the lock before connection for dialysis will enhance tolerance while the addition of enoxaparin will effectively prevent the risk of TC dysfunction.

## Methods/designs

### Aims

The purpose of ETERNITY is to assess the efficacy of an interdialytic lock solution (Ethenox) as compared with reference lock solutions (UFH 5000 U/mL or Citrate 4% *w*/*v*), for preventing TCIs in chronic hemodialysis patients.

### Ethical considerations and registrations

The study is promoted by the Centre Hospitalier Universitaire de Clermont-Ferrand. It was collaboratively designed by the dialysis unit, the medical intensive care unit and the department of clinical research and public health of the university hospital of Clermont-Ferrand, and Unit 6023 (Clermont-Ferrand, France) of the CNRS (French National Center for Scientific Research). The study complies with the principles of the Helsinki Declaration 2008. The whole protocol has been reviewed and approved by the Comité de Protection des Personnes—Sud-Est VI (no. 2016-AU1269). The investigator will explain the study to the eligible patient and give him/her the information form. Patients will be allowed the time they require to make a decision, after which the investigator will collect two initialed copies of the information form and two signed copies of the written consent form. The study is registered on Clinical Trials (NCT03083184) and on the European Clinical Trials Database (EudraCT 2016-A00180-51).

### Design and setting of the study

**Fig. 1:**
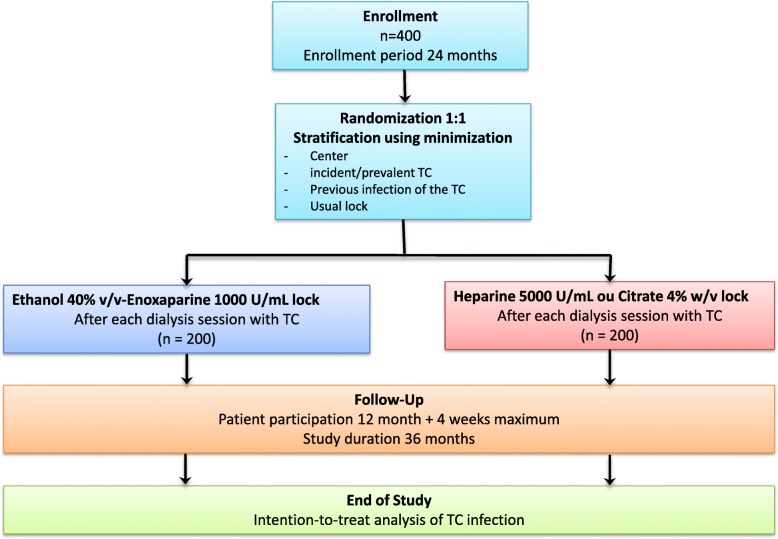
Schema for the Eternity trial. Enrolled patients will be randomly assigned in a 1:1 ratio either to the intervention group (Ethenox) or to the control group (reference solution: UFH 5000 U/mL or citrate 4% *w*/*v* depending on which solution is generally used). Random allocation will be performed by minimization using a computer algorithm. The study will be performed single blind for the patients and the analysts. The primary outcome will be time to first TCIs assessed by an endpoint adjudication committee blinded from study arm according to predefined criteria

**Fig. 2:**
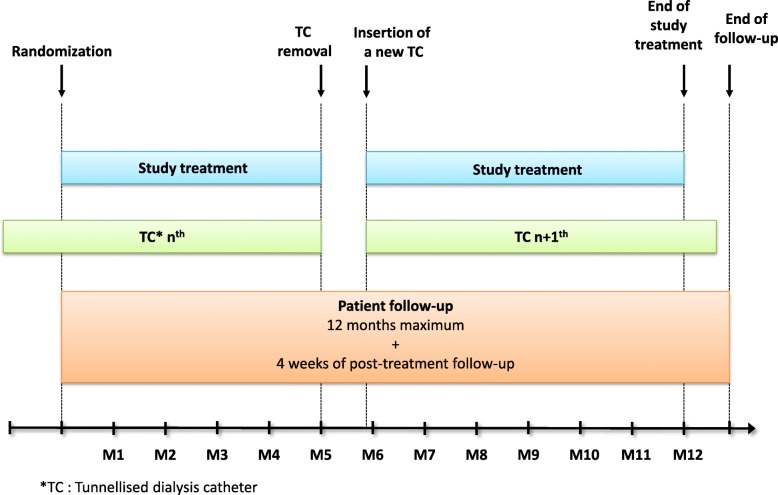
Scheme of participation in the study for a patient. TC is the statististical unit and several consecutive TC will be analysed in a same patient from the time the inclusion criteria are fullfilled

ETERNITY is a prospective phase III multi-centre, single blind, randomized, controlled, parallel group study (Fig. 1). It will be carried out in 14 dialysis units in France (Additional file 2). The planned duration of the study will be 36 months. The enrolment period will be 24 months. Patients will receive the study treatment for up to 12 months or until 1 month before the end of the study. To evaluate safety, all the patients included will be followed for adverse events for 4 additional weeks after they have received their last treatment dose in the study (Fig. 2).

### Study population

Eligible patients are adult (≥18 years of age) patients undergoing chronic hemodialysis in the study dialysis centres and treated with hemodialysis/haemodiafiltration via a TC inserted in a jugular vein three times a week for at least 2 weeks.

Exclusion criteria are intended use of the catheter for less than 3 months, intolerance of or hypersensitivity to ethanol, enoxaparin or UFH and citrate, previous history of type II heparin-induced thrombocytopenia, local or systemic antibiotic treatment for prevention or treatment of a TCI in the previous 2 weeks, presence of TCI in the previous 4 weeks, previous history of bacteraemia without a negative blood culture evidence of healing, TC impregnated with anti-infective agents or anticoagulants, pregnancy or risk of pregnancy (i.e. pre-menopausal woman not using a reliable method of contraception) and breastfeeding.

### Endpoints

#### Primary endpoints

The primary outcome will be time to first TCI. In the study, TCI is a composite endpoint defined by the occurrence of at least one of the three following events: definitive TCBSI, probable TCBSI or ESI. TCI diagnosis and its type (definitive or probable TCBSI or ESI) will be assessed by an endpoint adjudication committee (EAC, see Additional file 1) blinded to study arm according to predefined criteria (Tables 1 and 2).

**Table 1:** TC infections are defined according to the definitions of the guidelines of the DIALIN^6^ network

*TC exit-site infection*	
• Purulent discharge from the exit-site *Or* • Other local signs: redness or pain at the exit-site or along the subcutaneous tunnel (tunnellitis) *And at least one of the following criteria* - Fever (> 38.2 °C) *Or* - Positive blood culture drawn from one of the two lumens of the TC following aspiration of the lock *Or* - Polymorphonuclear leucocytosis In the absence of criterion for TC-related bloodstream infection

**Table 2:** The definition of TCBSI is specified according to K/DOQI 2006 clinical practice guidelines for vascular access^57^ which distinguishes between definitive and probable TCBSI

*Definitive TC- related bloodstream infection*	*Probable TC-related bloodstream infection*
At least one positive blood culture drawn from a peripheral vein or the arterial line up to 48 h after TC removal if performed *And at least one of the following criteria:* Positive quantitative broth culture of TC tip (Brun Buisson technique) > 10^2^ UFC/mL with the same microorganism *Or* Positive culture of the exit-site discharge with the same microorganism *Or* Positive blood culture drawn from one of the lumen of the TC following aspiration of the lock with the same microorganism and with a concentration ratio > 3 or a differential time to positivity > 2 h in relation to the blood culture sampled from the peripheral vein *Or* Resolution of signs of infection within 48 h following TC removal with no other apparent source of sepsis other than the TC	At least one positive blood culture drawn from a peripheral vein or the arterial line for a pathogenic microorganism (*S. aureus, enterobacteriaceae*, *pseudomonas sp., Candida sp*.) *Or* At least two positive blood cultures from a peripheral vein or the arterial line *Or* At least one positive blood culture from a peripheral vein or the arterial line in an immunocompromised patient Up to 48 h after TC removal if performed No other apparent source of sepsis other than the TC

#### Secondary endpoints

Secondary endpoints will comprise:

1. Other TCI prevention criteria
  Time to first definitive or probable TCBSI
  Time to first ESI
  Incidence rate of definitive or probable TCBSI
  Incidence rate of ESI
2. Prevention of TC colonizations: rates of colonization of removed TCs
3. Catheter patency
  Time to first TC dysfunction
  Incidence rate of TC dysfunctions
  Dialysis dose: monthly (Kt/V)_sp_ and (KT/V)_e_ (2nd generation Daugirdas equation) and Kt/V measured by the dialysis machine (ionic dialysance or optical measurement of urea in the effluent dialysate)
4. TC survival: time to TC removal
5. Health care consumption
  Time to first systemic antibiotic treatment for TCI
  Total duration and type of antibiotic treatment
  Time to first hospitalization for TCI
  Total duration of hospital stays for TCI
  Total number of TCs replaced during the study
6. Safety
  Incidence rate of breaches in TC integrity (TC leakage or disruption)
  Incidence rate of clinical adverse events related to ethanol exposure (tiredness, ethanol taste, headaches, dizziness, nausea, light-headedness, and increase in serum aspartate aminotransferase, alanine aminotransferase, gamma-glutamyl transferase or alkaline phosphatase).
  Incidence rate of type II heparin-induced thrombocytopenia.
  Incidence rate of haemorrhages: bleeding or suspected bleeding, qualified as major if associated with a decrease in haemoglobin levels of more than 2 g/dL or the need to transfuse at least two units of red blood cells.
7. Overall mortality: time to death from any cause
8. Inflammation: CRP value (measured every 3 months).

### Description of the products

Ethenox solution is obtained by mixing a pre-packaged ethanol-saline solution with 0.6 mL of enoxaparin (10,000 U/mL) to achieve 6 mL of the required ethanol content and enoxaparin concentration (enoxaparin 1000 U/mL in 40% *v*/v ethanol). The pharmacy department of the university hospital of Clermont-Ferrand will prepare the ethanol-saline solution in flasks and will send them with enoxaparin syringes every 3 months.to the study centres in boxes containing 14 treatments.

UFH lock solution is pure sodium heparinate 5000 U/mL packaged in the form of a single-use ampoule, i.e. heparin 25,000 U / 5 mL. Citrate solution at 4% is packaged in the form of a single-use ampoule, i.e. sodium citrate 4% 5 mL.

Each centre is free to choose the antiseptic solution used but cannot change it during the course of the study. The use of an antiseptic ointment at the exit-site until wounds have healed or in the event of ESI is authorized in accordance with guidelines [26]. The use of a fibrinolytic lock solution is not allowed except in the event of TC dysfunction. The use of other antimicrobial locks is not allowed and nor is that of dressings impregnated with an antibiotic or antiseptic.

### Study description

#### Screening

Patients eligible to participate in the study will be identified by a clinical research assistant in each study centre at the beginning of the study and then weekly.

#### Enrolment

Eligible patients will be recruited during a routine hemodialysis session by an investigator from centres participating in the study. The investigator will explain the study to the patient and give him/her the information form. The patients willing to participate in the study will sign the written consent form.

#### Randomization

Enrolled patients will be randomly assigned in a 1:1 ratio either to the intervention group (Ethenox) or to the control group (reference solution: UFH 5000 U/mL or citrate 4% *w*/*v* depending on the solution instilled in the patient’s TCs prior to inclusion). Random allocation will be performed by minimization using a computer algorithm. Minimization strata will be the study centre, the incident or prevalent nature of the TC and in the prevalent group the existence or not of a previous infection of the TC in place (Fig. 1).

#### Blinding

The recognisable smell of ethanol and the need to prepare the Ethenox lock solution makes it difficult to ensure the blinding of lock solutions by nurses despite the use of masks. The study will be performed single blind for the patients and the analysts.

#### Study intervention

##### TC handling protocol

TC will be handled according to the protocol established previously in each dialysis centre in compliance with the recommendations of the French Society for Hospital Hygiene [27]. In particular, the dressing of the TC must be changed systematically at least once a week or when the dressing is no longer occlusive or in the event of signs of TCI (shivering, pain caused by the catheter or fever). The use of TCs is exclusively reserved for renal replacement therapy. Outside the dialysis centre, apart from emergency procedures, it is prohibited to use TCs for the infusion of solution or for blood sampling. If this were to occur, the patient will be included only in the intent-to-treat (ITT) analysis.

##### Treatment under study

Ethenox in the intervention group and reference solutions in the control group will be used by the hemodialysis nurse as TC lock solution after each hemodialysis session with all TCs inserted in patients during the study period. After the patient is disconnected from the hemodialysis machine, the lumens of the TC will be rinsed with 20 mL of NaCl solution at 0.9%. The lock solution under study (Ethenox, UFH or Citrate 4% *w*/*v*) will be instilled slowly with a syringe into each lumen of the TC. The volume injected corresponds to the intraluminal volume of the TC branch determined at the time of inserting the TC according to its length (Canaud TC) or as inscribed on the catheter (palindrome TC). The lumens of the TC are then blocked with a sterile plug and the occlusive dressing of the TC is performed. The lock is left in place until the next hemodialysis session. The interdialytic lock (Ethenox or control) will be aspirated before connection at the start of the following hemodialysis session according to the routine procedure of the centres.

The physician in charge of the patient will suspend or discontinue the study treatment if he/she considers that the patient’s health requires it. Patients who will not be administered their study lock more than once a month will be nevertheless included in the per-protocol analysis. In the event of temporary change of dialysis centre (hospitalization, holiday, etc.) the study lock will be suspended and resumed when the patients return to their dialysis centre. These patients will be included only in the ITT analysis.

##### Conduct to be followed in the event of signs of TCI

In patients presenting signs of TCI, the lock studied will be maintained and patient management will be implemented according to the decision taken by the physician responsible for the patient in accordance with the practice of each centre. The diagnosis of TCI and its type will be confirmed or not a posteriori by the EAC.

##### Conduct to be followed in the event of catheter dysfunction

At each dialysis session, when extracorporeal blood flow of ≥300 ml/min cannot be achieved with an arterial line pressure higher than − 300 mmHg, the strategy to follow for restoring catheter patency will be to use successively, if necessary, flushing of catheter lumens with saline using a 10 mL syringe, reposition of the patient, reversal of the lines, and fibrinolytic therapy in the absence of contra-indications (administered, for example, at the start of the following dialysis session). The value of blood flow rate and pressure on the arterial line just before each reversal of the lines or fibrinolytic therapy prescription will be noted on the nurse’s record. The time of reversal of the line or fibrinolytic therapy use will be noted on the nurse’s record. Except when a fibrinolytic interdialytic lock is prescribed, the study lock will be maintained.

##### Conduct to be followed in the event of protocol violation

Anticipated violations to study protocol have been described above. Violations not mentioned can be recorded by the CRA as required by the protocol. Any questions regarding protocol violations should be directed to the steering committee (Additional file 1) by phone.

#### Study assessment

At each session, the nurse in charge of the patient will search for signs indicating the presence of a TCI or dysfunction using a check list (comprising clinical signs of infection and bacteriological samples, antibiotic treatment, TC lines inversion and fibrinolytic treatments) according to the usual practice of the dialysis centres and clinical practice guidelines [26, 28]. Data will be collected weekly in an electronic case report form specifically developed for the study. Data for adjudication of the primary outcomes and serious adverse events will be recorded continuously.

### Organization of the trial

#### Funding/support

ETERNITY is sponsored by the university hospital of Clermont-Ferrand (CHU de Clermont Ferrand, Clermont-Ferrand, France) and supported by a grant from the French Ministry of Health (Programme Hospitalier de Recherche Clinique National 2015, Study sponsor code: PHRC N 2015 ANIORT).

#### Coordination and conduct of the trial

Before the start of the study, all health-care workers and clinical research assistants in the 14 participating study centres will attend a formal training session on the study protocol, patient randomization and data collection in the Clinsight Clinical Data Monitoring Software (CDMS) electronic case report form. CDMS is a secure, interactive, web response system available at each study centre. The data manager will have access to the dataset. He will send login with limited access to each investigators ans local clinical research assistant. All documents required for the study will be available in each dialysis centre. Data monitoring in each center will be made by the same coordinating clinical research assistant independent from each study center. The principal investigator and a clinical research assistant will be available to discuss any problem concerning data collection, treatment invoices and protocol compliance and to assess study progress. Informations relative to important protocol modifications will be communicated to investigators using email and CDMS.

#### Interim analysis and independent data and safety monitoring committee

An independent data and safety monitoring committee (Additional file 1) will analyse the adverse events, give its opinion on major amendments of the protocol, proceed to the interpretation of an intermediate analysis (at 1/3 of the inclusions) and if deemed necessary announce the suspension or early termination of the study.

### Sample size calculation

The working hypothesis of this randomized controlled trial is that Ethenox will double the median time to TCI. On the basis of the data collected in DIALIN, median TC duration use is 6 months [29]. The median survival time without TCI reported in recent clinical trials ranges between 690 and 1150 days. However, to achieve a statistical power of 90% with a risk of type I error of 5%, a total of 400 patients need to be enrolled over a period of 24 months.

### Statistical analysis

#### General consideration

The statistical unit of the primary analysis and of most secondary analyses will be the TC. When a new TC is inserted in a patient participating in the study, the new TC will be analysed for the occurrence of a TCI or TC dysfunction only from the time when the patient fulfils the eligibility criteria. This will rule out ongoing TCI or early dysfunction of the TC due to poor positioning at the time of insertion or an anatomical abnormality of the patient.

The principal analysis will be performed on the intention-to-treat principle with Stata software (version13, StataCorp, College Station, TX). The tests will be two-sided, with a type I error set at α = 0.05. Quantitative variables will be presented as mean ± SD when normally distributed (assumption of normality studied by Shapiro-Wilk test), and for non-normal distributions as median, quartiles and range. Qualitative variables will be expressed as numbers and associated percentages.

#### Primary analysis

To evidence the efficacy of the Ethenox lock solution in preventing TCIs in comparison with the reference lock solutions (UFH 5000 U/mL or Citrate 4%), the censored data will be estimated by the Kaplan-Meier method. The comparison between groups regarding the primary endpoint will be performed with a marginal Cox proportional hazard model. Results will be expressed as hazard ratios and 95% confidence interval. Second, owing to the possible non-random censoring process the proposition to right-censor patients at the time of TC removal is debatable. The previous models will be performed with the Inverse Censoring Probability Weighting estimator. Finally, because of possible competing events, which prevent an event of interest from occurring rather than just preventing it from being seen to happen (censoring), Fine and Gray’s approach will be used.

#### Secondary analysis

For the secondary endpoint, the censored data will be estimated by the Kaplan-Meier method and compared between groups by a marginal Cox proportional hazard model. Concerning the parameters collected longitudinally, mixed models will be performed to take into account inter- and intra-patient variability. The effects of group, time and their interaction (time x group) will be studied as fixed effects, with the patient effect considered as a random effect (data repeated for the same subject). Lastly, regarding comparisons for which the statistical unit will be the patient, the usual tests will be applied: Student t-test or Mann-Whitney test if the conditions of the t-test are not competed (normality, homoscedasticity studied by the Fisher-Snedecor test) for parameters of a quantitative nature and the Chi-squared test or Fisher’s exact test for categorical variables.

#### Method of taking into account missing and invalid data

Regarding the longitudinal data, a sensitivity analysis will be performed to study the attrition bias to ascertain the quantity (level of attrition) and nature (independence of the study group) of missing data to propose the most appropriate method of data imputation such as that of Verbeke and Molenberghs.

## Discussion

TC use is frequent in chronic hemodialysis patients [30, 31]. Thus, prevention of TCIs is essential. TCIs lead to systemic antibiotic administration in more than 70% of cases [29]. This contributes to an increase in the prevalence of multi-resistant pathogens. Decreasing TCI rates by the use of Ethenox solution will reduce antibiotic consumption and the emergence of multi-resistant pathogens.

TCIs are responsible for high morbidity and mortality in chronic hemodialysis patients [1]. TCBSI leads to hospitalization for 75% of patients. Mortality related to TCBSI is about 20%. The harmful effects of TCIs are also indirect. Recurrent TCIs are associated with a chronic low-grade inflammatory state [32] that is involved in the pathogenesis of protein-energy wasting syndrome. TCIs have also been associated with an increased risk of cardio-vascular events [33]. Thus, inflammation due to infection may cause accelerated atherosclerosis. Prevention of TCI is therefore of paramount importance for improving endpoints in chronic hemodialysis patients fitted with TCs.

Ethenox solution is less expensive than the other antimicrobial solutions (€3.20 for Ethenox made with enoxaparine as against €30 for taurolidine citrate) and of equal cost as citrate 4% *w*/*v* (€2.80). The price of enoxaparin will decrease once enoxaparin generics, which are already on the market in several countries, are made available in France. Several studies have estimated the cost of treating a TCBSI at about €20,000 [34]. Thus, preventing TCI could lead to a significant reduction in health expenditure.

Finally, the results of this study will help to establish with a high level of evidence national and international recommendations for the prevention of TCIs in chronic hemodialysis patients.

## Additional files

Additional file 1: Members of the steering committee, endpoint adjudication committee and data and safety monitoring committee. (DOCX 14 kb)

Additional file 2: Study centers. List of the dialysis units/hospital that are involved in this study. (DOCX 13 kb)

## Abbreviations

ESI: Exit-site infection
ITT: Intention to treat
LMWH: Low molecular weight heparins
TC: Tunnelled dialysis catheter
TCBSI: TC-related bloodstream infections
TCI: TC infection
UFH: Unfractionated heparin
