# Supplementary material for: Evaluation of the efficacy of an interdialytic “ethanol 40% v/v - enoxaparin 1000 U/mL” lock solution to prevent tunnelled catheter infections in chronic hemodialysis patients: a multi-centre, randomized, single blind, parallel group study
Source: BMC Nephrol. 2019 Apr 30;20:149. doi: 10.1186/s12882-019-1338-6 (PMC6492371; doi:10.1186/s12882-019-1338-6)
Supplement: Supplementary file 1 — Members of the steering committee, endpoint adjudication committee and data and safety monitoring committee. (DOCX 14 kb) [file 12882_2019_1338_MOESM1_ESM.docx]

**Additional file 1**

**Steering committee**

**Dr Julien ANIORT**

Néphrologue, Service de Néphrologie, Dialyse et Transplantation rénale

CHU de Clermont-Ferrand

58 Rue Montalembert 63003 CLERMONT-FERRAND

[janiort@chu-clermontferrand.fr](mailto:janiort@chu-clermontferrand.fr)

**Pr Anne-Elisabeth HENG**

Néphrologue, Service de Néphrologie, Dialyse et Transplantation rénale

CHU de Clermont-Ferrand

58 Rue Montalembert 63003 CLERMONT-FERRAND

[aheng@chu-clermontferrand.fr](mailto:aheng@chu-clermontferrand.fr)

**Dr Hélène LERAY MORAGUES**

Néphrologue, Service de Néphrologie, Dialyse et Transplantation rénale

CHU Lapeyronie

191, avenue du doyen Gaston Giraud 34295 Montpellier cedex

[h-leray_moragues@chu-montpellier.fr](mailto:h-leray_moragues@chu-montpellier.fr)

**Pr Bertrand SOUWEINE**

Réanimateur médical, Unité de Réanimation Médicale Polyvalente

CHU de Clermont-Ferrand – Hôpital Gabriel Montpied

58 Rue Montalembert 63003 CLERMONT-FERRAND

[bsouweine@chu-clermontferrand.fr](mailto:bsouweine@chu-clermontferrand.fr)

## **Endpoint adjudication committee**

**Dr Julien ANIORT**

Néphrologue, Service de Néphrologie, Dialyse et Transplantation rénale

CHU de Clermont-Ferrand

58 Rue Montalembert 63003 CLERMONT-FERRAND

[janiort@chu-clermontferrand.fr](mailto:janiort@chu-clermontferrand.fr)

**Dr Olivier BAUD**

Infectiologue, Service d’Hygiène Hospitalière

CHU de Clermont-Ferrand

58 Rue Montalembert 63003 CLERMONT-FERRAND

[obaud@chu-clermontferrand.fr](mailto:obaud@chu-clermontferrand.fr)

**Dr Marc BOUILLER**

Néphrologue, Service de Néphrologie, Dialyse et Transplantation rénale

CH du Puy en Velay, Hôpital Emile Roux

12, bd du Docteur Chantemesse - BP 20352 43012 Le Puy en Velay

[marc.bouiller@ch-lepuy.fr](mailto:marc.bouiller@ch-lepuy.fr)

**Pr Bertrand SOUWEINE**

Réanimateur médical, Unité de Réanimation Médicale Polyvalente

CHU de Clermont-Ferrand – Hôpital Gabriel Montpied

58 Rue Montalembert 63003 CLERMONT-FERRAND

[bsouweine@chu-clermontferrand.fr](mailto:bsouweine@chu-clermontferrand.fr)

## **Data and safety monitoring committee**

**Pr. Jean-François TIMSIT**

APHP – Hôpital Bichat

UMR 1137 IAME Equipe 5 Decision Sciences In Infectious Diseases

Université Paris Diderot, Paris, France

[jean-francois.timsit@bch.aphp.fr](mailto:jean-francois.timsit@bch.aphp.fr)

**Pr Kada KLOUCHE,**

néphrologue et réanimateur médical, Réanimation métabolique et UARP Lapeyronie

CHU Lapeyronie

191, avenue du doyen Gaston Giraud 34295 Montpellier

[k-klouche@chu-montpellier.fr](mailto:k-klouche@chu-montpellier.fr)

**Dr Jean Ralph ZAHAR,**

infectiologue, Département biologie des agents infectieux et pharmaco-toxicologie -Unité de prévention et Lutte contre les infections nosocomiale (UPLIN)

CHU d’Angers

4 Rue Larrey, 49100 Angers

[jrzahar@gmail.com](mailto:jrzahar@gmail.com).
