# Supplementary material for: Evaluation of the efficacy of an interdialytic “ethanol 40% v/v - enoxaparin 1000 U/mL” lock solution to prevent tunnelled catheter infections in chronic hemodialysis patients: a multi-centre, randomized, single blind, parallel group study
Source: BMC Nephrol. 2019 Apr 30;20:149. doi: 10.1186/s12882-019-1338-6 (PMC6492371; doi:10.1186/s12882-019-1338-6)
Supplement: Supplementary file 2 — Study centers. List of the dialysis units/hospital that are involved in this study. (DOCX 13 kb) [file 12882_2019_1338_MOESM2_ESM.docx]

**Study centers**

| Center | Address |
| --- | --- |
| CH du Puy en Velay | CH du Puy en Velay, Hôpital Emile Roux,  12 bd du Docteur Chantemesse BP 20352 43012 Le Puy en Velay |
| AGDUC Grenoble | AGDUC Grenoble  Chemin de l'agnelas  BP 41 CHU 38700 LA TRONCHE |
| CH Chambéry | Centre hospitalier de Chambéry,  Place Lucien Biset 73011 Chambéry |
| Calydial, Vienne | Calydial, Site de Vienne, CH Lucien Hussel  Montée du Dr Chapuis, 38209 VIENNE CEDEX |
| CHU de Clermont-Ferrand | CHU de Clermont Ferrand, Hôpital Gabriel Montpied, 7 place Henri Dunant 63000 Clermont-Ferrand |
| CHU Lyon HEH | Unité d’hémodialyse, Hôpital Edouard Herriot,  Place d'Arsonval, 69437 Lyon |
| CHU de Strasbourg | Service de Néphrologie et Dialyse Nouvel Hôpital Civil, 1 place de l’Hôpital 67091 Strasbourg cedex |
| CHU Montpellier | Service de Néphrologie-Transplantation-Soins Intensifs,  Hôpital Lapeyronie  34295 Montpellier Cedex 05 |
| AIDER | Centre de dialyse d’Alès, Association Installation à Domicile Epurations Rénales (AIDER)  414 Chemin des Potences, 30100 Alès |
| CHU Saint-Etienne | Service de Néphrologie, Dialyse et Transplantation rénale  Hôpital Nord, CHU de St-Etienne,  42055 SAINT-ETIENNE cedex 2 |
| CH Perpignan | Service de Néphrologie-  Centre Hospitalier de Perpignan-Hôpital Saint Jean- BP 4052  66046 Perpignan Cedex |
| Médipôle Saint-Roch | Centre de néphrologie Polyclinique Médipôle Saint-Roch,  Rue Ambroise Croizat, 66330 Cabestany |
